# Supplementary material for: Identification of photoperiod-induced specific miRNAs in the adrenal glands of Sunite sheep (Ovis aries)
Source: Front Vet Sci. 2022 Jul 22;9:888207. doi: 10.3389/fvets.2022.888207 (PMC9354845; doi:10.3389/fvets.2022.888207)
Supplement: Supplementary file 1 [file Data_Sheet_1.ZIP › Supplementary file/miRDeep2 file.zip!/miRDeep2 file/Novel_Structure/SPLP42A2/Novel_395.pdf]

Provisional ID	: NC_019464.2_17112
Score total	: 2.3
Score for star read(s)	: -1.3
Score for read counts	: 0
Score for mfe	: 2
Score for randfold	: 1.6
Score for cons. seed	:
Total read count	: 12
Mature read count	: 12
Loop read count	: 0
Star read count	: 0

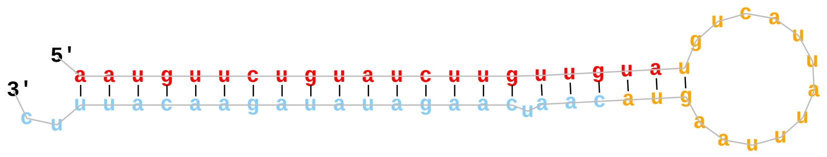

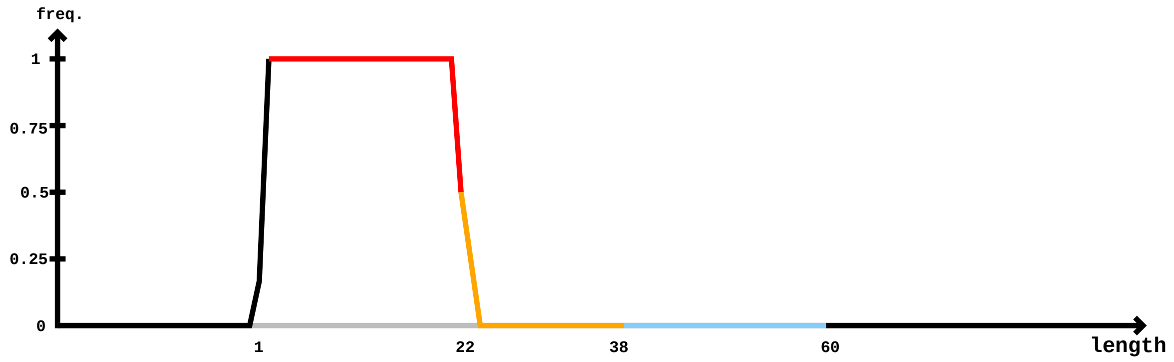

Mature Star

```
5'- ggaaaccuggggguaauggaaauguucuguaucuuguuguaugucauuauuuaaguacaaucaagauauagaacauuuccauuacacucaagcuuaauuaaauggauauau   -3'    exp
    .......((((.((((((((((((((((((((((((((((((............)))))).))))))))))))))))))))))))).)))).....................     reads    mm    sample
    ...................aaauguucuguaucuuguugu.......................................................................     2        0     seq
    ....................aauguucuguaucuuguugu.......................................................................     4        0     seq
    ....................aauguucuguaucuuguugua......................................................................     6        0     seq
```
